# Supplementary material for: First-order spatial coherence measurements in a thermalized two-dimensional photonic quantum gas
Source: Nat Commun. 2017 Jul 31;8:158. doi: 10.1038/s41467-017-00270-8 (PMC5537358; doi:10.1038/s41467-017-00270-8)
Supplement: Supplementary file 1 — Supplementary Information [file 41467_2017_270_MOESM1_ESM.pdf]

File Name: Supplementary Information
Description: Supplementary Figures, Supplementary Notes and Supplementary References.

File Name: Peer Review File
Description:

Supplementary note 1: Introduction

In this Supplementary information, we give a theoretical study of the spatial and temporal first-order correlations of a two-dimensional photon gas confined in a harmonic trapping potential, assuming thermal equilibrium conditions. First, a rigorous analytical calculation for the first-order correlations of a classical (uncondensed) photon gas is presented. To test for the analytical model with its prediction for the transverse coherence length and the longitudinal coherence time, also numerical calculations are conducted. The numerical methods furthermore allow to extend predictions of the coherence properties to the Bose-Einstein condensed phase, where an analytical evaluation of the first-order correlation function fails. We investigate the scaling of the spatial and temporal first-order correlation length as a function of the total particle number and temperature, respectively. In the classical regime below the onset of Bose-Einstein condensation, the degree of first-order coherence is Gaussian, showing short-range correlations which decay on a length scale determined by the thermal de Broglie wavelength λ_{th}, see e.g. refs. 1,2. Our numerical results further verify the expected temperature dependence of $\lambda_{\mathrm{th}} \propto 1/\sqrt{T}$ for the transverse coherence length and $\tau_{\mathrm{c}} \propto 1/T$ for the longitudinal coherence time. In the quantum degenerate regime of the photon gas in the microcavity, when the lowest energy state is macroscopically occupied, our numerical results show the emergence of long-range correlations. The experimental observations presented in the main text are well described by the here discussed theory model.

Supplementary note 2: Theory of spatial correlations

In this section, we calculate the first-order spatial correlations of a thermalized two-dimensional photon gas at temperature T in a harmonic potential of angular trapping frequency Ω, for total particle numbers well below the critical particle number for Bose-Einstein condensation $N_{\mathrm{c}} = \pi^2/3\,(k_{\mathrm{B}}T/\hbar\Omega)^2$. To describe the experiments, we here theoretically study the first-order correlations $G^{(1)}(\mathbf{r},\mathbf{r}')$ between two transverse positions $\mathbf{r}$ and $\mathbf{r}'$ in the photon gas at the same point in time. Accordingly, we will omit the explicit time dependence of the photon fields for the calculation of the transverse first-order correlations.

In the harmonic trapping potential the field operators $\hat{\Psi}$ and $\hat{\Psi}^\dagger$ can be expanded in harmonic oscillator eigenfunctions $\psi_n(\mathbf{r}) = \psi_n^*(\mathbf{r})$ and operators $\hat{a}_n, \hat{a}_n^\dagger$ for creation and annihilation, respectively, of a photon in an eigenstate with index n

$$\hat{\Psi}(\mathbf{r}) = \sum_m \psi_m(\mathbf{r})\hat{a}_m, \quad \text{and} \quad \hat{\Psi}^\dagger(\mathbf{r}) = \sum_n \psi_n^*(\mathbf{r})\hat{a}_n^\dagger. \tag{1}$$

The general expression for the first-order correlation function can be obtained from a Fourier transformation of the Wigner function, providing a connection between quantum-mechanical correlation function and statistical mechanics, see e.g. ref [1]. This yields

$$G^{(1)}(\mathbf{r},\mathbf{r}') = \sum_n \psi_n^*(\mathbf{r})\,\psi_n(\mathbf{r}')\,\langle \hat{a}_n^\dagger \hat{a}_n\rangle = \sum_n \psi_n^*(\mathbf{r})\psi_n(\mathbf{r}')\,\bar{n}_n, \tag{2}$$

where we have used the orthogonality condition $\langle \hat{a}_m^\dagger \hat{a}_n\rangle = \delta_{mn}$ and the Bose-Einstein distributed average occupation of the n-th mode with energy ϵ_n at a chemical potential μ (regarding the two-

fold polarization degeneracy per mode)

$$\langle \hat{a}_n^\dagger \hat{a}_n \rangle = \bar{n}_n = \frac{2}{e^{(\epsilon_n - \mu)/k_\mathrm{B}T} - 1}. \tag{3}$$

In the dye microcavity the photon dynamics is restricted to the transverse degrees of freedom and discretized by the transverse modal quantum numbers n_x and n_y. The energy levels of the two-dimensional harmonic oscillator are given by $E_{n_x n_y} = \hbar\omega_c + \hbar\Omega(n_x + n_y + 1)$, where $\hbar\omega_c$ is the cavity cutoff energy. With respect to this low-energy cutoff, the transverse energies in eq. (3) can be renormalized

$$\epsilon_{n_x n_y} := E_{n_x n_y} - \hbar\omega_\mathrm{c} - \hbar\Omega = \hbar\Omega(n_x + n_y). \tag{4}$$

The corresponding spatially inhomogeneous eigenfunctions of the two-dimensional harmonic oscillator are given by

$$\psi_{n_x n_y}(x,y) = \frac{1}{\sqrt{2^{n_x} n_x!}} \frac{1}{\sqrt{2^{n_y} n_y!}} \left(\frac{m\Omega}{\pi\hbar}\right)^{1/2} e^{-\frac{m\Omega}{2\hbar}\left(x^2+y^2\right)} H_{n_x}\left(\sqrt{\frac{m\Omega}{\hbar}}x\right) H_{n_y}\left(\sqrt{\frac{m\Omega}{\hbar}}y\right), \tag{5}$$

with m as the photon mass and Hermite polynomials $H_n(x)$. The first-order correlation function then becomes

$$G^{(1)}(x,y,x',y') = \sum_{n_x,n_y} \frac{1}{2^{n_x} n_x!} \frac{1}{2^{n_y} n_y!} \frac{m\Omega}{\pi\hbar} e^{-\frac{m\Omega}{2\hbar}\left(x^2+x'^2+y^2+y'^2\right)} \frac{2}{e^{\left(\epsilon_{n_x n_y}-\mu\right)/k_B T} - 1}$$
$$\times H_{n_x}\left(\sqrt{\frac{m\Omega}{\hbar}}x\right) H_{n_x}\left(\sqrt{\frac{m\Omega}{\hbar}}x'\right) H_{n_y}\left(\sqrt{\frac{m\Omega}{\hbar}}y\right) H_{n_y}\left(\sqrt{\frac{m\Omega}{\hbar}}y'\right) \tag{6}$$

In the thermal phase, where the chemical potential is well below thermal energy, $\mu \ll -k_B T$, the photon number distribution follows a Boltzmann distribution $\bar{n}_{n_x,n_y} \simeq 2\, e^{\frac{\mu}{k_\mathrm{B}T}} e^{-\frac{\hbar\Omega}{k_\mathrm{B}T}(n_x+n_y)}$. In this case the correlation function at two identical positions $\mathbf{r} = (x,y)$ and $\mathbf{r}' = (x,y)$ simplifies to

$$G^{(1)}(x,y,x,y) = \frac{2m\Omega}{\pi\hbar} e^{\frac{\mu}{k_\mathrm{B}T}} \times \left[e^{-\frac{m\Omega}{\hbar}x^2} \sum_{n_x} \frac{e^{-\frac{\hbar\Omega}{k_\mathrm{B}T}n_x}}{2^{n_x} n_x!} H_{n_x}\left(\sqrt{\frac{m\Omega}{\hbar}}x\right)^2 \right]$$
$$\times \left[e^{-\frac{m\Omega}{\hbar}y^2} \sum_{n_y} \frac{e^{-\frac{\hbar\Omega}{k_\mathrm{B}T}n_y}}{2^{n_y} n_y!} H_{n_y}\left(\sqrt{\frac{m\Omega}{\hbar}}y\right)^2 \right]. \tag{7}$$

Using the parameters $A = \frac{2m\Omega}{\pi\hbar} e^{\frac{\mu}{k_\mathrm{B}T}}$, $\xi = \sqrt{\frac{m\Omega}{\hbar}}x$, $\eta = \sqrt{\frac{m\Omega}{\hbar}}y$, $\zeta = e^{-\frac{\hbar\Omega}{k_\mathrm{B}T}}$ we rewrite eq. (7) as

$$G^{(1)}(\xi,\eta,\xi,\eta) = A \left[e^{-\xi^2} \sum_{n_x} \frac{\zeta^{n_x}}{2^{n_x} n_x!} H_{n_x}(\xi)^2 \right] \times \left[e^{-\eta^2} \sum_{n_y} \frac{\zeta^{n_y}}{2^{n_y} n_y!} H_{n_y}(\eta)^2 \right]. \tag{8}$$

In analogy, the correlation function for remote positions $r = (x, y)$ and $r' = (x', y')$ follows:

$$G^{(1)}(\xi,\eta,\xi',\eta') = A\left[e^{-\frac{\xi^2+\xi'^2}{2}}\sum_{n_x}\frac{\zeta^{n_x}}{2^{n_x}n_x!}H_{n_x}(\xi)H_{n_x}(\xi')\right]\times\left[e^{-\frac{\eta^2+\eta'^2}{2}}\sum_{n_y}\frac{\zeta^{n_y}}{2^{n_y}n_y!}H_{n_y}(\eta)H_{n_y}(\eta')\right]. \quad (9)$$

By employing the relation from ref. 3,

$$e^{-(\xi^2+\eta^2)}\sum_n\frac{\zeta^n}{2^n n!}H_n(\xi)H_n(\eta) = \frac{1}{\sqrt{1-\zeta^2}}e^{-\frac{\xi^2+\eta^2-2\xi\eta\zeta}{1-\zeta^2}}, \quad (10)$$

we from eqns. (8) and (9) obtain

$$G^{(1)}(\xi,\eta,\xi,\eta) = A\times e^{\xi^2+\eta^2}\frac{1}{1-\zeta^2}e^{-\frac{2\left(\xi^2+\eta^2\right)}{1+\zeta}} \quad (11)$$

$$G^{(1)}(\xi,\eta,\xi',\eta') = A\times e^{\frac{\xi^2+\xi'^2+\eta^2+\eta'^2}{2}}\frac{1}{1-\zeta^2}e^{-\frac{\xi^2+\xi'^2-2\xi\xi'\zeta}{1-\zeta^2}}\times e^{-\frac{\eta^2+\eta'^2-2\eta\eta'\zeta}{1-\zeta^2}}. \quad (12)$$

The normalized degree of first-order spatial coherence immediately follows to be

$$g^{(1)}(\xi,\eta,\xi',\eta') = \frac{G^{(1)}(\xi,\eta;\xi',\eta')}{\sqrt{G^{(1)}(\xi,\eta;\xi,\eta)\times G^{(1)}(\xi',\eta';\xi',\eta')}} \quad (13)$$

$$= \exp\left[-\frac{\zeta}{1-\zeta^2}\left((\xi-\xi')^2+(\eta-\eta')^2\right)\right]. \quad (14)$$

By resubstituting the above defined parameters, one obtains the degree of first-order coherence of a thermalized classical two-dimensional photon gas

$$g^{(1)}(x,y,x',y') = \exp\left[-\pi\frac{(\mathbf{r}-\mathbf{r}')^2}{\lambda_{\mathrm{th}}^2}\right], \quad (15)$$

with the thermal de Broglie wavelength $\lambda_{\mathrm{th}} = \hbar\sqrt{2\pi/mk_{\mathrm{B}}T}$. This analytical result is exactly reproduced by our numerical calculations, see Supplementary Figure 1, which fulfill the same conditions as the experiment: the photon emission transmitted through one of the microcavity mirrors is split up and recombined with a retro-reflected copy of itself with inverted coordinates with respect to the origin, $(x, y) \leftrightarrow (-x, -y)$. With $\mathbf{r}' = -\mathbf{r}$ we obtain the degree of first-order coherence

$$g^{(1)}(r) = \exp\left[-\frac{4\pi r^2}{\lambda_{\mathrm{th}}^2}\right]. \quad (16)$$

Our numerical calculations (Supplementary Figure 1) are based on a Bose-Einstein distributed spectrum of photon energies and thus can be performed for different values of the chemical potential. In particular, this allows for a full numerical evaluation of the first-order correlations in the condensed phase for a large range of condensate fractions between 0% and close to 100%. The emergence of long-range order around the BEC threshold is visible in the two-dimensional maps showing $g^{(1)}(x, y, -x, -y)$ in Supplementary Figure 1(a), while Supplementary Figure 1(b) gives four corresponding spatial intensity distributions. Horizontal cuts through the correlation and intensity distributions at $y = 0$ are shown in Supplementary Figure 2(a). Below the critical particle number

the spatial extent of the thermal correlations is in very good agreement with the analytical result in eq. (16), i.e. the correlations decay on a length scale determined by the thermal de Broglie wavelength of the photons at room temperature. On the other hand, in the Bose-Einstein-condensed phase our theory predicts an enhancement of the spatial first-order correlations to larger extensions, see also Supplementary Figure 2(c) for data of the transverse correlation length versus condensate fraction. In the thermal phase, the short-range correlation length exhibits a temperature dependence, as shown in Supplementary Figure 2(b), which can be understood from a temperature dependence of the size of the thermal de Broglie wavelength.

Supplementary note 3: Theory of temporal correlations

In this section, the first-order temporal correlations of a trapped two-dimensional photon gas at thermal equilibrium conditions are calculated. As described above, the photons are confined in a harmonic trapping potential and the frequency of the photons in the transversal cavity modes is given by $E_{00}/\hbar = \omega_{\mathrm{c}} + \Omega\,(n_x + n_y + 1)$. For the sake of simplicity, we consider correlations of fixed positions $(x, y) = (x', y')$ at different times t, t', i.e. at non-vanishing time delays $\tau = t' - t \neq 0$,

$$G^{(1)}(x,y;t,t') = \sum_{n_x,n_y} \psi^*_{n_x n_y}(x,y) e^{-i[\omega_c+\Omega(n_x+n_y+1)]t} \psi_{n_x,n_y}(x,y) e^{i[\omega_c+\Omega(n_x+n_y+1)](n_x+n_y)t'} \bar{n}_{n_x,n_y}. \tag{17}$$

Far below the critical particle number, the first-order correlations of the thermal photon gas are

$$\begin{aligned} G^{(1)}(x,y;t,t+\tau) &= \frac{2m\Omega}{\pi\hbar} e^{\frac{\mu}{k_B T}} \sum_{n_x,n_y} \frac{1}{2^{n_x} n_x!\, 2^{n_y} n_y!} H_{n_x}\left(\sqrt{\frac{m\Omega}{\hbar}}x\right)^2 H_{n_y}\left(\sqrt{\frac{m\Omega}{\hbar}}y\right)^2 && (18)\\ &\quad \times e^{-\frac{m\Omega}{\hbar}(x^2+y^2)} e^{i\Omega(n_x+n_y)\tau - \frac{\hbar\Omega}{k_B T}(n_x+n_y)} e^{i(\omega_c+\Omega)\tau} && (19)\\ &= A e^{i(\omega_c+\Omega)\tau}\left[e^{-\xi^2}\sum_{n_x}\frac{\zeta^{n_x}}{2^{n_x} n_x!} H_{n_x}(\xi)^2\right] \times \left[e^{-\eta^2}\sum_{n_y}\frac{\zeta^{n_y}}{2^{n_y} n_y!} H_{n_y}(\eta)^2\right] && (20) \end{aligned}$$

Similar to Section 1 of this Supplementary Information, we have used $A := \frac{2m\Omega}{\pi\hbar} e^{\frac{\mu}{k_B T}}$, $\xi := \sqrt{\frac{m\Omega}{\hbar}}x$, $\eta := \sqrt{\frac{m\Omega}{\hbar}}y$ and a redefined parameter $\zeta(\tau) := e^{i\Omega\tau - \frac{\hbar\Omega}{k_B T}}$, for which we will in the following steps drop the explicit notation of its time dependence, i.e. $\zeta(\tau) \equiv \zeta$. Using eq. (10) then yields

$$G^{(1)}(x,y;t,t+\tau) = A\, e^{i(\omega_c+\Omega)\tau} e^{\xi^2+\eta^2} \frac{1}{1-\zeta^2} e^{-\frac{2\left(\xi^2+\eta^2\right)}{1+\zeta}} \tag{21}$$

To obtain the first-order correlation function at equal times, we set $\tilde{\zeta} := e^{-\frac{\hbar\Omega}{k_B T}}$, where the dependence on the time delay τ has dropped out, and find

$$G^{(1)}(x,y;t,t) = G^{(1)}(x,y;t',t') = A\, e^{\xi^2+\eta^2} \frac{1}{1-\tilde{\zeta}^2} e^{-\frac{2\left(\xi^2+\eta^2\right)}{1+\tilde{\zeta}}}. \tag{22}$$

This readily yields the degree of first-order temporal coherence at a fixed position

$$g^{(1)}(x,y;t,t') = \frac{G^{(1)}(x,y;t,t')}{\sqrt{G^{(1)}(x,y;t,t)G^{(1)}(x,y;t',t')}} \tag{23}$$

$$= e^{i(\omega_c+\Omega)\tau}\frac{1-\tilde{\zeta}^2}{1-\zeta^2}\exp\left[-2\left(\xi^2+\eta^2\right)\left(\frac{\tilde{\zeta}-\zeta}{(1+\zeta)\left(1+\tilde{\zeta}\right)}\right)\right]. \tag{24}$$

By resubstituting the parameters and using $r^2 = x^2 + y^2$ we obtain

$$g^{(1)}(r;\tau) = e^{i(\omega_c+\Omega)\tau}\ \frac{1-e^{-2\frac{\hbar\Omega}{k_BT}}}{1-e^{-2\frac{\hbar\Omega}{k_BT}+2i\Omega\tau}}\exp\left[-\frac{2m\Omega}{\hbar}r^2\left(\frac{1-e^{i\Omega\tau}}{1+e^{\frac{\hbar\Omega}{k_BT}}+e^{i\Omega\tau}+e^{-\frac{\hbar\Omega}{k_BT}+i\Omega\tau}}\right)\right]. \tag{25}$$

In the following, we omit the spatial dependence and consider the center of the photon gas at $r = 0$ only. Neither our experiments nor the ideal Bose gas theory indicate non-trivial propagating correlations, as were observed e.g. in polariton systems (see ref. 4). The first term in eq. (25) then determines the complex valued degree of first-order temporal coherence

$$g^{(1)}(\tau) = \frac{\left(e^{2\frac{\hbar\Omega}{k_BT}}-1\right)\left\{\cos\left[(\omega_c+\Omega)\tau\right]+i\sin\left[(\omega_c+\Omega)\tau\right]\right\}}{e^{2\frac{\hbar\Omega}{k_BT}}-\cos(2\Omega\tau)-i\sin(2\Omega\tau)}. \tag{26}$$

In the experiment one measures the real part of eq. (26)

$$\mathrm{Re}\left[g^{(1)}(\tau)\right] = \frac{\left(e^{2\frac{\hbar\Omega}{k_BT}}-1\right)\left\{e^{2\frac{\hbar\Omega}{k_BT}}\cos\left[(\omega_c+\Omega)\tau\right]-\cos\left[(\omega_c-\Omega)\tau\right]\right\}}{1+e^{4\frac{\hbar\Omega}{k_BT}}-2e^{2\frac{\hbar\Omega}{k_BT}}\cos(2\Omega\tau)}, \tag{27}$$

which exhibits an oscillatory behaviour due to constructive and destructive interference between the photons from both arms of the Michelson interferometer at the detector. The degree of first-order coherence $\mathrm{Re}\left\{g^{(1)}(\tau)\right\}+1$ is plotted in Supplementary Figure 3(a) as a function of the time delay for 4 different temperatures $T = \{100, 300, 380, 1000\}$K. The absolute of eq. (26) corresponds to the visibility shown in Supplementary Figure 3(b)

$$|g^{(1)}(\tau)| = \frac{\left(1-e^{-2\frac{\hbar\Omega}{k_BT}}\right)}{\sqrt{\left[1-e^{-2\frac{\hbar\Omega}{k_BT}}\cos(2\Omega\tau)\right]^2+\left[e^{-2\frac{\hbar\Omega}{k_BT}}\sin(2\Omega\tau)\right]^2}}, \tag{28}$$

which determines the coherence time τ_c as the time when the visibility has decayed to 0.5. With $a := \cosh\left(\frac{\hbar\Omega}{k_BT}\right)$ and $b := \sinh\left(\frac{\hbar\Omega}{k_BT}\right)$ it can be expressed by

$$\tau_c = \frac{1}{\Omega}\arccos\left\{\frac{\sqrt{-3+10a^2-3a^4-20ab+12a^3b+10b^2-18a^2b^2+12ab^3-3b^4}}{2(a-b)}\right\}. \tag{29}$$

As the inverse thermal excitation number $\hbar\Omega/k_\mathrm{B}T \approx 0.007$ is well below unity in the dye microcavity,

an expansion up to first order in $\hbar\Omega/k_\mathrm{B}T$ provides a solution for the first-order correlation time

$$\tau_c \simeq \sqrt{3}\frac{\hbar}{k_\mathrm{B}T}\,. \tag{30}$$

Hence, the first-order (longitudinal) correlation time of the thermal photon gas is expected to scale as $1/T$, whereas the (transverse) spatial correlation length is expected to scale as $1/\sqrt{T}$, as given in eq. (16).

In the previously discussed derivation of the first-order temporal correlations of the thermal photon gas, the classical Boltzmann approximation $\bar{n}_{n_x n_y} \propto \exp\left(-\epsilon_{n_x n_y}/k_\mathrm{B}T\right)$ was used to obtain an analytical solution for $g^{(1)}(\tau)$. At the onset of Bose-Einstein condensation however, when the transverse ground mode occupation becomes macroscopic with respect to the total photon number, the first-order temporal correlations are significantly enhanced and the correlation function cannot be evaluated analytically any more. In this regime, the numerical calculations can be performed without restrictions and provide the exact first-order temporal correlation function by retaining the full Bose-Einstein distribution $\bar{n}_{n_x n_y} \propto \left[\exp\left(-\epsilon_{n_x n_y}/k_\mathrm{B}T\right) - 1\right]^{-1}$. Supplementary Figure 4 shows the visibility $|g^{(1)}(\tau)|$ at the center of the photon gas for 7 different values of the chemical potential and the total photon number, respectively, when crossing over from the thermal to the condensed regime at a temperature of 300K. Far below criticality, see the two lowest curves in Supplementary Figure 4, the coherence time of $\tau_c = 44\,\mathrm{fs}$ shows excellent agreement with the analytical result from eq. (30). As the total photon number is increased the coherence time becomes longer and above the critical particle number exceeds the investigated time range of 650fs.

Supplementary note 4: Interference experiment

In our experiment, the spatial and temporal correlations of the photon gas are studied by directing the light that is transmitted through one of the cavity mirrors into a Michelson-type interferometer, which allows both for a variation of the transverse displacement and the temporal delay between the interfering paths. One arm of the interferometer is equipped with a plane mirror, while the other arm hosts a cat-eye retroreflector mounted onto a linear motorized translation stage, with the latter spatially inverting the position coordinates of the photons. Both beam paths are recombined on an EMCCD camera with a quantum efficiency of approximately 97%. The spatial resolution of the used optical system is roughly $0.93\,\mu$m, as we have verified by placing a point-like optical source (SNOM fiber, 200 nm diameter) in the microcavity plane, where the thermal photon gas is generated. In this section, we will briefly summarize the expected spatio-temporal intensity profile at the position of the detector.

The quantized photon field operators in eq. (1) can be extended by temporally varying phase terms

$$\hat{\Psi}(\mathbf{r}', t') = \sum_m \psi_m(\mathbf{r}') e^{-\frac{i}{\hbar}E_m t'} \hat{a}_m, \quad \text{and} \quad \hat{\Psi}^\dagger(\mathbf{r}, t) = \sum_n \psi_n^*(\mathbf{r}) e^{\frac{i}{\hbar}E_n t} \hat{a}_n^\dagger. \tag{31}$$

Correspondingly, the first-order correlation function at a temporal delay $\tau = t' - t$ due to a difference

in the interferometer arm lengths becomes

$$G^{(1)}(\mathbf{r},\mathbf{r}';\tau)=\langle\hat{\Psi}^{\dagger}(\mathbf{r},t)\hat{\Psi}(\mathbf{r}',t')\rangle=\sum_{k}\psi_k^{*}(\mathbf{r})\psi_k(\mathbf{r}')e^{-\frac{i}{\hbar}E_k\tau}\,\bar{n}_k. \quad (32)$$

As the spatial intensity distribution of the photon gas in the microcavity, see ref. 5, is given by

$$I(\mathbf{r})\simeq\frac{\hbar\omega_c}{\tau_{rt}}\sum_{k}|\psi_k(\mathbf{r})|^2\bar{n}_k=\frac{\hbar\omega_c}{\tau_{rt}}G^{(1)}(\mathbf{r},\mathbf{r};0)=\frac{1}{2}\varepsilon_0 c\langle|E(\mathbf{r},t)|^2\rangle, \quad (33)$$

one can make the heuristic ansatz for the operator of the electric field

$$\hat{E}(\mathbf{r},t)=\sqrt{\frac{2\hbar\omega_c}{\varepsilon_0 c\tau_{rt}}}\hat{\Psi}(\mathbf{r},t)=\sqrt{\frac{2\hbar\omega_c}{\varepsilon_0 c\tau_{rt}}}\sum_{m}\psi_m(\mathbf{r})e^{-\frac{i}{\hbar}E_m t}\hat{a}_m. \quad (34)$$

Assuming that the cavity emission of spatial intensity distribution $I(\mathbf{r})$ is incident on a symmetric beam splitter with $|r_i|^2=|t_i|^2=\frac{1}{2}$, the superposition of the electric fields in both the reflecting and spatially inverting interferometer arms results in an intensity at position $\mathbf{r}$ at the detector

$$\begin{aligned}I_d(\mathbf{r},\tau)&=\frac{1}{2}\varepsilon_0 c\langle|\frac{1}{2}E(\mathbf{r},t)+\frac{1}{2}E(-\mathbf{r},t')|^2\rangle\\&=\frac{1}{8}\varepsilon_0 c\frac{2\hbar\omega_c}{\varepsilon_0 c\tau_{rt}}\left\{\sum|\psi_m(\mathbf{r})|^2\bar{n}_m+\sum|\psi_m(-\mathbf{r})|^2\bar{n}_m+2\,\mathrm{Re}\left[\sum_m\psi_m^{*}(\mathbf{r})\psi_m(-\mathbf{r})\,e^{-\frac{i}{\hbar}E_m\tau}\bar{n}_m\right]\right\}\\&=\frac{1}{4}\left\{I(\mathbf{r})+I(-\mathbf{r})+2\sqrt{I(\mathbf{r})I(-\mathbf{r})}\;\mathrm{Re}\left[g^{(1)}(\mathbf{r},-\mathbf{r};\tau)\right]\right\} \quad (35)\end{aligned}$$

Here $\langle ...\rangle$ denotes a temporal averaging over times which are long compared to oscillation period of the optical field ($\tau=\lambda_c/c\simeq 2\,\mathrm{fs}$, for $\lambda_c=583\,\mathrm{nm}$) caused by the detector and the degree of first-order coherence is given by

$$g^{(1)}(\mathbf{r},-\mathbf{r};\tau)=\frac{\langle\hat{E}^{\dagger}(\mathbf{r},t)\hat{E}(-\mathbf{r},t')\rangle}{\sqrt{\langle\hat{E}^{\dagger}(\mathbf{r},t)\hat{E}(\mathbf{r},t)\rangle}\sqrt{\langle\hat{E}^{\dagger}(-\mathbf{r},t)\hat{E}(-\mathbf{r},t)\rangle}}=\frac{\sum_m\psi_m^{*}(\mathbf{r})\psi_m(-\mathbf{r})\,e^{-\frac{i}{\hbar}E_m\tau}\bar{n}_m}{\sqrt{\sum_m|\psi_m(\mathbf{r})|^2\bar{n}_m}\sqrt{\sum_m|\psi_m(-\mathbf{r})|^2\bar{n}_m}}.$$

The real part of $g^{(1)}(\mathbf{r},-\mathbf{r};\tau)$ varies like an amplitude-modulated cosine function with an in general complicated phase $\Delta\phi(\omega_m,k_m)$, accounting for the thermal distribution of eigenfrequencies and a potential wavevector mismatch in both arms $(k_m-k'_m)x$. Our final expression for the spatial intensity distribution at the output of the Michelson interferometer after rewriting eq. (35) is

$$I_d(\mathbf{r},\tau)=\frac{1}{4}\left\{I(\mathbf{r})+I(-\mathbf{r})+2\sqrt{I(\mathbf{r})I(-\mathbf{r})}\,|g^{(1)}(\mathbf{r},-\mathbf{r};\tau)|\,\cos\left[\Delta\phi(\omega_m,k_m)\right]\right\}. \quad (36)$$

The absolute of the degree of first-order coherence thus determines the visibility of the interference fringes, which are measured in the experiment:

$$V(\mathbf{r},\tau)=\frac{I_{\max}-I_{\min}}{I_{\max}+I_{\min}}=\frac{2\sqrt{I(\mathbf{r})I(-\mathbf{r})}}{I(\mathbf{r})+I(-\mathbf{r})}|g^{(1)}(\mathbf{r},-\mathbf{r};\tau)| \quad (37)$$

Supplementary note 5: Effects of finite spatial resolution

The finite spatial resolution of the optical imaging system used in the experiment modifies the measured correlation signal, which has to be accounted for in the numerical calculations. Assuming a radially symmetric intensity distribution $I(\mathbf{r}) = I(-\mathbf{r})$, the real part and the absolute of the degree of first-order temporal coherence at any position $\mathbf{r}$ read

$$\mathrm{Re}\left[g^{(1)}(\mathbf{r},\tau)\right] = \frac{4I_d(\mathbf{r},\tau)}{2I(\mathbf{r})} - 1 \quad \text{and} \quad |g^{(1)}(\mathbf{r},\tau)| = V(\mathbf{r},\tau), \tag{38}$$

which give the degree of temporal coherence at an infinitesimal spatial position. Experimentally however one measures the intensity $\tilde{I}_d(\mathbf{r},\tau)$, which is averaged over the vicinity of the position under investigation. The width of the averaging is determined by the spatial resolution of the imaging apparatus; more precisely, it is quantified by the width of the point spread function on the detector produced by a point-like source placed in the resonator plane, where the thermal photon gas is generated. Numerically, this can be accounted for by integrating over the nearby detection area at each given time delay,

$$\begin{aligned} \tilde{I}_d(\mathbf{r},\tau) &= \frac{1}{A}\int_{\mathrm{PSF}} I_d(\mathbf{r}',\tau)\exp\left[-\frac{(\mathbf{r}-\mathbf{r}')^2}{2\sigma_{\mathrm{PSF}}^2}\right] d\mathbf{r}' \\ &\simeq \frac{I(\mathbf{r},\tau)}{2}\left\{1+\frac{1}{A}\int_{\mathrm{PSF}} |g^{(1)}(\mathbf{r}',-\mathbf{r}';\tau)|\exp\left[-\frac{(\mathbf{r}-\mathbf{r}')^2}{2\sigma_{\mathrm{PSF}}^2}\right] d\mathbf{r}'\right\}, \end{aligned}$$

with the normalization factor $A = \int_{\mathrm{PSF}} \exp\left[-\frac{(\mathbf{r}-\mathbf{r}')^2}{2\sigma_{\mathrm{PSF}}^2}\right] d\mathbf{r}'$. Due to its slow variation with position, $I(\mathbf{r},\tau)$ can be considered constant over a length scale given by the standard deviation σ_{PSF} of the point spread function, and thus it is excluded from the integral. Finally, one obtains the following expected experimentally detectable degree of first-order coherence

$$|\tilde{g}^{(1)}(\mathbf{r},-\mathbf{r};\tau)| = \frac{2\tilde{I}_d(\mathbf{r},\tau)}{I(\mathbf{r},\tau)} - 1. \tag{39}$$

The numerical result for the real part of the spatially averaged first-order correlation $\mathrm{Re}\left[g^{(1)}(0,0;\tau)\right]$ for an uncondensed, thermal photon gas with $\mu \simeq -7.0k_{\mathrm{B}}T$ is shown in Supplementary Figure 5(a). The data demonstrates that the visibility of the averaged interference fringes (green line) is reduced at short time delays, as compared to the visibility of the interference fringes with perfect spatial resolution (blue line). At large time delays, the absolute of the averaged first-order correlation function approaches the non-averaged visibility, as remote points with $|\mathbf{r}| > 0$ here exhibit no significant contributions due to the finite spatial coherence of the photons. Similarly, when approaching the condensed phase regime (see Supplementary Figure 5(b) for three increasing values of the chemical potential), no significant difference between the averaged (solid line) and non-averaged (thick solid line) visibility is observed, as is well understood from the here long-range spatial correlations which by far exceed the imaging resolution. By integrating the numerically calculated interference signals over an area equivalent to that of the point spread function, the numerical results well reproduce our experimentally measured signals observations (grey lines in Supplementary Figure 5(a,b)). The

finite imaging resolution has to be particularly considered if one measures the spatial correlations at zero path difference in the interferometer ($\tau \simeq 0$). Supplementary Figure 5(c) shows the analytical result for the first-order spatial correlations (thick blue line) from eq. (16), the numerical result with (dashed line) and without (red line) a convolution with the experimentally determined point spreading function with $\sigma_{\mathrm{PSF}} = 0.658(2)\,\mu$m, along with the experimental data (yellow circles). The experimentally observed data are in excellent agreement with the theoretical averaged correlation function.

Supplementary note 6: Effects of detector characteristics

Our experimental procedure to obtain two-dimensional maps of spatial correlations relies on a position-resolved measurement of the fringe visibility in an interferometer with nearly equal arm lengths. As visible e.g. in Figure 1(c) of the main text, the density of the harmonically trapped photon gas is not spatially homogeneous, but rather due to the effect of the trapping potential concentrated around the trap center. Correspondingly, while the visibility of the interference pattern can be determined reliably in this dense central region, a determination of the interference contrast in the low-intensity regions is much more imprecise. The finite noise floor of the used camera now in the outer spatial regions with a small signal turns out to be additive, as our camera always gives only positive signal outputs, while the effect of the noise is both positive and negative for a large camera signal, as obtained near the trap center. For illustration purposes, Supplementary Figure 6 shows a cut through the camera signal recorded in the absence of a fringe pattern, which shows the described effect of (asymmetric) symmetric noise in the (low intensity) high intensity region, respectively. For the data with a fringe pattern, the presence of the asymmetric noise floor of the camera δI in the outer parts of the photon cloud with $I_{\mathrm{max,min}} \to I_{\mathrm{max,min}} + \delta I$ effectively reduces the visibility value V_{exp} of the interference fringes derived from the observed camera signals,

$$V(\mathbf{r}) = \frac{I_{\mathrm{max}} - I_{\mathrm{min}}}{I_{\mathrm{max}} + I_{\mathrm{min}}} =: \frac{A}{B}, \quad V_{\mathrm{exp}}(\mathbf{r}) = \frac{A}{B + 2\delta I}. \tag{40}$$

An expansion in $2\delta I$ yields

$$V_{\mathrm{exp}}(\mathbf{r}) = V(\mathbf{r}) \left[1 - \frac{\delta I}{\bar{I}(\mathbf{r})} + \left(\frac{\delta I}{\bar{I}(\mathbf{r})}\right)^2 - \left(\frac{\delta I}{\bar{I}(\mathbf{r})}\right)^3 + ... \right] = V(\mathbf{r}) \left(1 + \frac{\delta I}{\bar{I}(\mathbf{r})}\right)^{-1}, \tag{41}$$

with $I_{\mathrm{max}} + I_{\mathrm{min}} \approx 2\bar{I}(\mathbf{r})$, where $\bar{I}(\mathbf{r})$ denotes the average intensity in the vicinity of position $\mathbf{r}$. While for large intensities the derived value for the visibility approaches the ideal value, for low intensity levels the obtained visibility value is significantly reduced. From a measurement of the spatial intensity distribution, we find significant deviations between experimental and theoretical data for photon densities near $3 \cdot 10^{13}\,\mathrm{m}^{-2}$, see Supplementary Figure 6. The effect of this detector noise floor has been accounted for in the numerical model calculation of the expected fringe contrast. The corresponding expected curves can be found in Figures 4(b),(c) of the main text, which results in a much better agreement with the experimentally determined visibility values.

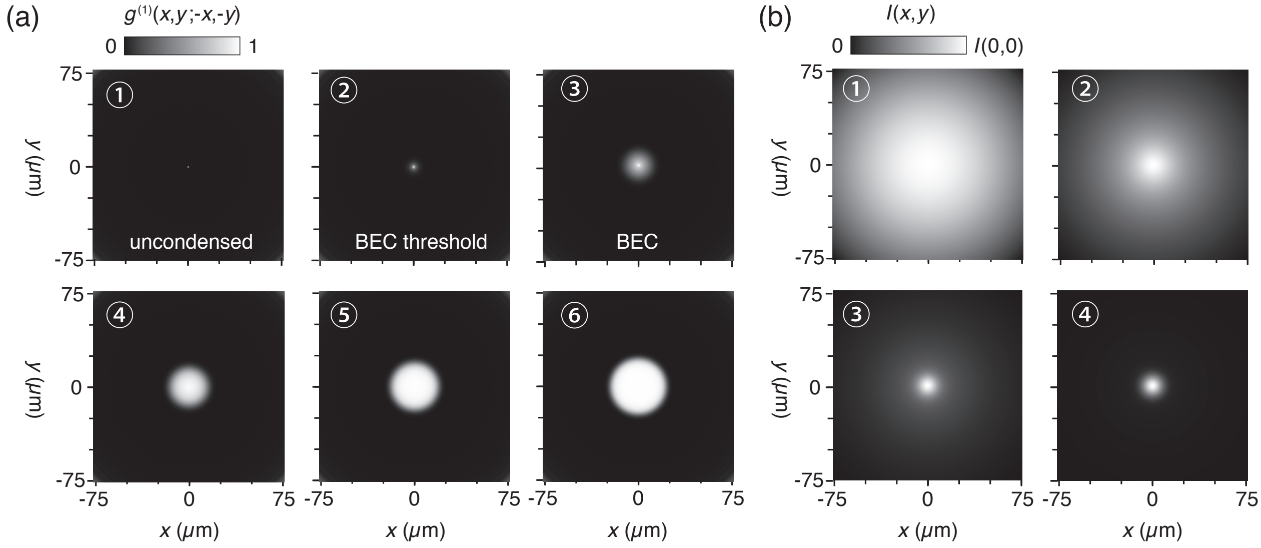

Supplementary Figure 1: **Numerical results for correlations and intensity distributions.** (a) First-order spatial correlations $g^{(1)}(x, y; -x, y)$ for increasing values of the chemical potential $\mu_{①\text{-}⑥} = -\{10^0; 10^{-2}; 10^{-3}; 10^{-4}; 10^{-5}; 10^{-6}\}\, k_B T$ (relative ground state occupation of $(n_0/N)_{①\text{-}⑥} = \{0.01\%; 0.2\%; 2\%; 19\%; 70\%; 96\%\}$) when crossing over from the thermal to the Bose-Einstein condensed phase. The chemical potential at criticality ($N_c = 94\,000$) is given by approximately $\mu_② \simeq -0.01\, k_B T$. In the thermal phase, correlations are short-range and determined by the thermal de Broglie wavelength. In the condensed phase, full spatial coherence is established within the condensate mode volume. (b) Spatial intensity distributions of the photon gas for corresponding values of $\mu_{①\text{-}④}$, both below and above critical photon number. All numerical calculations include contributions from the first 700 energy levels.

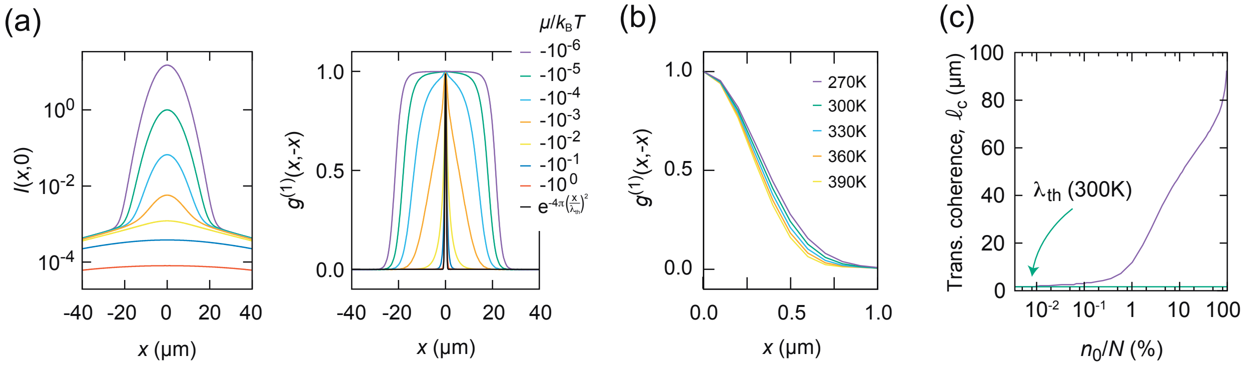

Supplementary Figure 2: **Spatial profiles of intensity distribution and first-order correlations.** (a) $I(x, 0)$ and $g^{(1)}(x, -x)$ along x-axis for different values of the chemical potential ranging from the thermal to the Bose-Einstein-condensed phase. The solid black line corresponds to a theory curve based on eq. (16). (b) Transverse spatial correlations of the thermal photon gas (with N = const.) for different temperatures. When the photon gas is heated up, its correlation length decreases due to a reduction of the thermal de Broglie wavelength of the photons. (c) Extension of the transverse coherence length (FWHM) versus condensate fraction. Below the critical photon number, the coherence decays on a length scale characteristic for the thermal de Broglie wavelength $\lambda_{th}(300\text{K}) = 1.48\,\mu m$, while the correlation length increases when the threshold for Bose-Einstein condensation is exceeded.

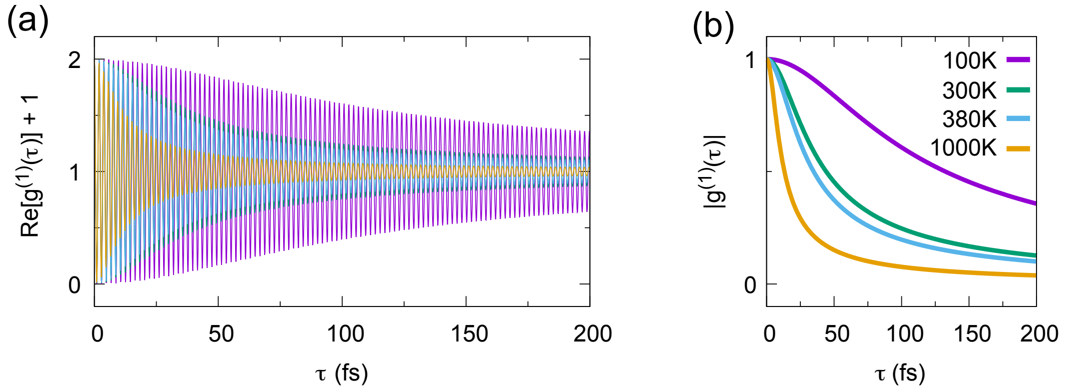

Supplementary Figure 3: **Temperature dependence of the degree of first-order temporal coherence.** (a) Real part of the degree of coherence as a function of time delay for 4 different temperatures based on the analytical derivation presented in this Supplementary Information. (b) Absolute of the degree of coherence, which corresponds to the visibility in an interference experiment with observed signal of type (a), for respective temperatures. For all plots, the photon gas is assumed to be in thermal equilibrium with a total particle number far below the critical number for Bose-Einstein condensation.

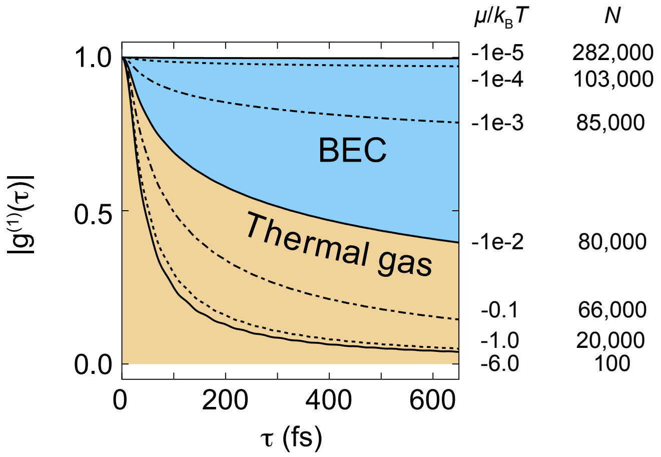

Supplementary Figure 4: **Numerical calculation of the degree of first-order temporal coherence for different photon numbers.** Far below condensation threshold (lowest two curves), the coherence decays on a time scale given by the analytical formula of eq. (30), which yields $\tau_c \simeq 44$ fs for 300K in perfect agreement with our numerics. With the onset of a Bose-Einstein condensate, long-range temporal coherence is established, which very soon above the threshold exceeds the shown time range of 650 fs. Note that above $\mu/k_{\mathrm{B}}T \simeq -10^{-2}$ one crosses from the thermal to the Bose-Einstein condensed phase. Besides the chemical potential also the corresponding total number of particles N is given.

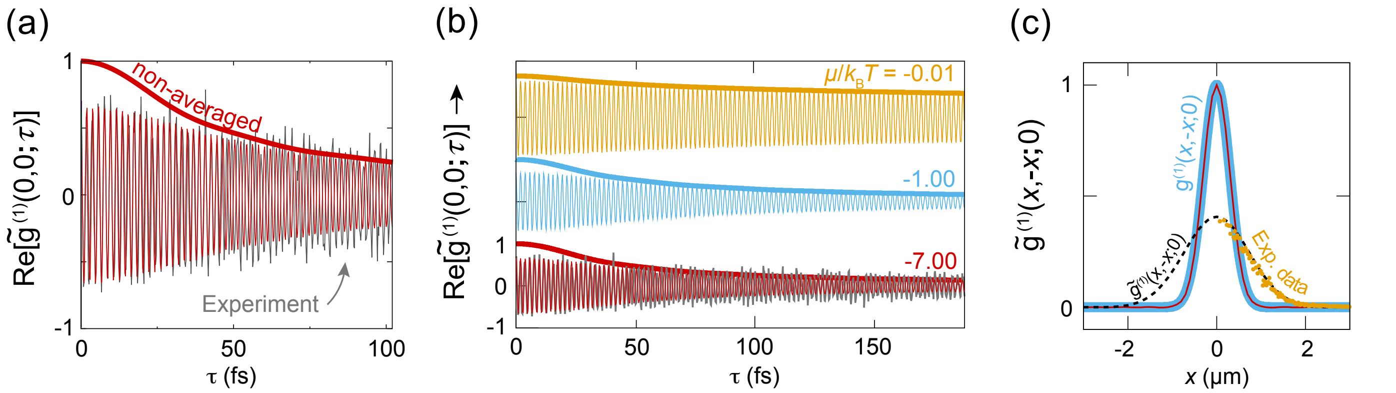

Supplementary Figure 5: **Effects of finite spatial resolution.** (a) Experimentally observed interference signal versus time delay (grey) along with numerically calculated interference signal (red) averaged over an area determined by the point spread function. The averaging leads to a reduction of the visibility of the interference fringes below the non-averaged visibility at the central position of the photon gas (thick red line). (b) Numerically calculated averaged interference signals versus time delay as the photon gas number is increased towards Bose-Einstein criticality. The effect of averaging is mostly visible at short time delays, and nearly vanishes as the chemical potential is increased towards criticality ($\mu/k_{\mathrm{B}}T \simeq -0.01$). (c) Spatial first-order correlations of a thermal photon gas at room temperature along the x-axis. The analytical result (blue) agrees with the numerical result (red). The finite imaging resolution in the experiment can be accounted for in the numerical calculations by convolving the spatial first-order correlation function with the point spread function (dashed line), demonstrating very good agreement between experiment and theory.

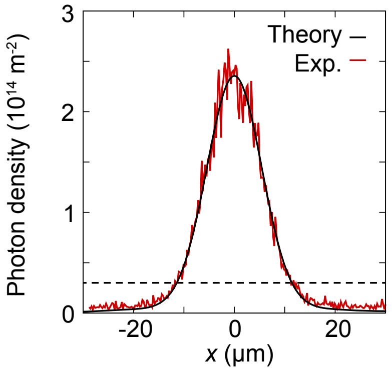

Supplementary Figure 6: **Detection noise floor.** Horizontal cut through the detected spatial intensity distribution from a single-shot measurement of a Bose-Einstein condensed photon gas in the absence of a fringe pattern (red line), along with theory for the photon density (black line). For photon densities below roughly $3 \cdot 10^{13}\,\mathrm{m}^{-2}$ (dashed line) the experimental data reveals a noise floor which exceeds the expected photon distribution. For the data with a fringe pattern, these effects cause a correction to the derived fringe visibility, in contrast to the observation in the trap center with large signal, where the noise is both positive and negative.

Supplementary References

[1] Naraschewski, M. & Glauber, R. J. Spatial coherence and density correlations of trapped Bose gases. *Phys. Rev. A* **59**, 4595–4607 (1999).

[2] Hadzibabic, Z. & Dalibard, J. Two-dimensional Bose fluids: An atomic physics perspective. *Riv. Nuovo Cimento* **34**, 389 (2011).

[3] Sakurai, J. J. *Modern Quantum Mechanics* revised edn (Addison-Wesley, 1994).

[4] Roumpos, G. et al. Power-law decay of the spatial correlation function in exciton-polariton condensates. *Proc. Natl. Acad. Sci.* **109**, 6467–6472 (2012).

[5] Klaers, J., Schmitt, J., Vewinger, F. & Weitz, M. Bose-Einstein condensation of photons in an optical microcavity. *Nature* **468**, 545–548 (2010).
